# Characterization of compulsory hospitalization in the psychiatry department of Faro

**DOI:** 10.1192/j.eurpsy.2021.1083

**Published:** 2021-08-13

**Authors:** M. Viseu De Carvalho, F. Gomes Tavares, M. Barbosa Pinto, M. Mota Oliveira

**Affiliations:** 1 Psychiatry, Centro Hospitalar Universitário do Algarve, Faro, Portugal; 2 Psychiatry, Centro Hospitalar Universitário do Algarve - Unidade Faro, Faro, Portugal

**Keywords:** compulsory hospitalization, urgent compulsory regimen, compulsory treatment

## Abstract

**Introduction:**

The mental health law is not universal. In Portugal the urgent compulsory regimen is based on the principles of the presence of severe mental anomaly and risk to themselves or others.

**Objectives:**

Characterization of patients admitted in compulsory hospitalization (CH) between 2018 and 2020, in the University Hospital Center of Algarve - Faro Department

**Methods:**

Retrospective study from January/2018 to August/2020. Social, demographic, epidemiological and clinical data were evaluated.

**Results:**

Of 1191 patients who were admitted 36% occurred in CH. There was a predominance in male sex (58%), the average age was 43 years old. For 62% it wasn´t the first hospitalization and 58% had a recent prescription. Admission occurred most frequently because of the presence of psychotic symptoms (figure1) and according to the international classification of disease (ICD10) the most frequent diagnosis was unspecified nonorganic psychosis (figure2). At discharge 59% of the patients remained on compulsory treatment (CT), where the most frequent diagnosis was schizophrenia. Although most of these patients were discharged with oral and long-term injectable antipsychotics, there were higher rates of readmissions when compared to non-CT group, were the most frequent diagnosis was mental and behavioural disorders due to psychoactive substance use and most of the patients were discharged only with oral therapy.

**Figure:**
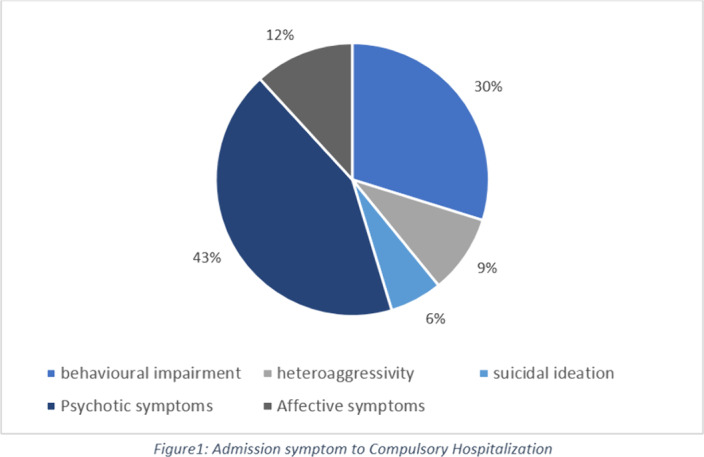

**Figure:**
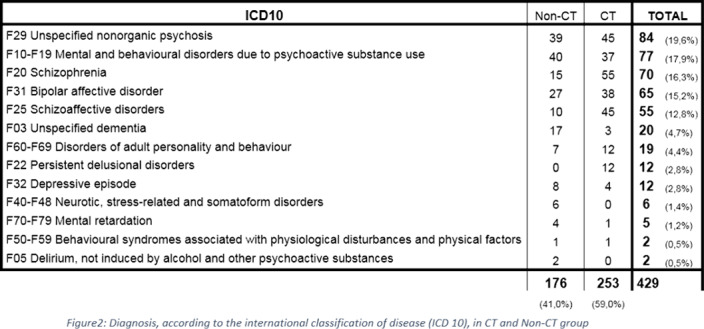

**Conclusions:**

Patients in compulsory regimen have a severe mental illness with higher rates of readmission. We highlight the use of long-term injectable antipsychotics in terms of compliance. These findings corroborate the need for specialized and multidisciplinary approach in terms of psychosocial rehabilitation in these patients.

